# Hospital and Institutional News

**Published:** 1916-06-10

**Authors:** 


### HOSPITAL AND INSTITUTIONAL NEWS.

### THE NEW CHELSEA HOSPITAL FOR WOMEN.

The new buildings of the Chelsea Hospital for Women, the plans of which, with a full description, were published in The Hospital of July 10, 1915, are rapidly approaching completion, and it has been announced that Her Majesty the Queen has graciously consented to open them in person on July 11. It is proposed in honour of this event to name a ward-block after Her Majesty; and an appeal is being issued for £10,000 in donations as an additional method of celebrating the occasion. This sum will not clear off the cost of the whole scheme, for which an additional £20,000 beyond the sum now appealed for will be required. The appeal is issued from Holland House, and is signed by Lord Londonderry, president of the hospital, and by Lady Ilchester, president of the ladies' committee.

### THE BIRTHDAY HONOURS.

From civilian and institutional standpoints the list of "Birthday Honours" is somewhat unexciting this year: the naval and military lists, however, make up in length and variety what the others lack. For the decorations and promotions conferred upon officers of the Medical Services reference should be made to the list which is published on page 238. Sir Savile Crossley, Bart., K.C.V.O., receives the dignity of a Barony; he has been since its foundation Honorary Secretary of King Edward's Hospital Fund for London. Mr. C. A. Pearson becomes a baronet; blind himself, his energy in work of all sorts on behalf of his fellow-sufferers has been untiring, especially for those who have lost their sight through wounds in the war. Dr. Addison, one of the few medical men in the House of Commons, is made a Privy Councillor. There are three knighthoods of medical or of institutional interest: Mr. G. P. Doolette, the West Australian gold magnate, is president of the Australian Voluntary Hospital at Wimereux; Professor Nestor Tirard was medical editor of the latest edition of the "British Pharmacopœia," and has been for many years physician to King's College Hospital; and Dr. A. M. Ruffer, C.M.G., is president of the Quarantine Board in Egypt. In the appointments to the Order of the Bath much the most interesting is that of the Hon. Arthur Stanley, M.V.O., M.P., to be Companion of the Order. Mr. Bernard Mallet, the Registrar-General, is promoted from Companion to Knight Commander. Practically the only other civilian decorations conferred are the M.V.O. of the fourth class on Dr. Bardswell, the superintendent of the King Edward VII. Sanatorium, Midhurst, and on Dr. Stanley Hewett, Surgeon-Apothecary to the King; and the Kaisar-i-Hind Gold Medal for public services in India awarded to a Punjab surgeon-missionary, Mr. H. M. Newton; to Dr. R. G. Robson, of Ajmer; and to Dr. Peter Cullen, of Jubbulpore, whose record shows the unusual combination of a retired lieut.-colonelcy of the Indian Medical Service with Holy Orders.

### THE TEACHING OF MEDICAL ETHICS.

At the recently concluded sessions of the General Medical Council a motion was passed requiring that in courses of instruction of medical students in forensic medicine and public health, or otherwise, the duties of practitioners in relation to the State and the generally recognised rules of medical ethics shall in future be taught. We have every sympathy with this proposal, the result of which should be very valuable if the right teachers are chosen to give the lectures in the medical schools. We can imagine with what fascination some of the more versatile teachers might handle this topic—Dr. F. J. Smith of "the London," for instance—and how exceedingly uninteresting most lecturers would make it. Two experts who could probably deal with the subject very fully and authoritatively are the secretaries of the two medical defence societies, for they have to cope so often with quarrels and disputes engendered by failure to appreciate and act up to the not very difficult code of medical ethics. The exact legal status of hospital residents and visiting staffs in relation to their boards and committees, as well as to secretaries, matrons, and other officials, is another branch of the subject which might well fill up one lecture, and the ethical relations of such medical officers to one another, to their patients, to nursing staffs, and to governing bodies, might well also be taken into consideration.

### WASTAGE OF ARMY DOCTORS.

The *Daily Telegraph*, which ventilated very freely last winter the views of that large body of Army doctors who believe there is grave and avoidable waste of medical men in the British Expeditionary Force, has returned to the attack in a very downright article by the medical correspondent of that paper. This article makes four definite suggestions with a view to economy of medical effort at the Front. These suggestions are somewhat revolutionary, but there is a lot to be said in favour of them, and we trust they will not be cold-shouldered with the usual official assurance that the present system is perfect. The first two are that field ambulances should be done away with, thus setting free about thirty doctors per division for more useful work; and that the medical unit should no longer be the division, but the army. Unquestionably such an alteration would be practicable, economical, and beneficial to all concerned so long as trench war continues. If trench war is to continue to the end of the campaign, as seems highly probable, this reform would go far to solve the shortage of medical officers, and would be in the best interests of the wounded too. It is perhaps unlikely to be adopted, as it would interfere a good deal with the "vested interests" of the R.A.M.C. The other suggestions are that doctors should not be employed on purely clerical work, and that it is wasteful to keep a full complement of doctors (doing nothing) in the field ambulances of the home defence army. The former suggestion is sound as far as it goes, and the latter principle is even more worthy of adoption. It would be interesting to know whether the Germans are using the division as their medical unit; most probably not, one would think.

### A CARNT MEMORIAL AT MANCHESTER.

We are glad to learn that the board of management of the Manchester Royal Infirmary has decided to name a ward after the late general superintendent, Mr. Walter G. Carnt. He was respected throughout the hospital world for his knowledge, practical experience, and administrative gifts, and liked for the ready way in which he was invariably willing to place them at the disposal of the numerous inquirers and correspondents who came in search of friendly hints from him. The record of his work was published in The Hospital of September 4, 1915, and, as his name will always be associated with the successful inauguration of the great new infirmary, it is only fitting that one of the wards should bear his name. We ourselves should have liked to see the name of Walter Carnt associated with the next development that the Royal Infirmary undertakes, for he was fully alive to the fact that new departures always await even the best-equipped and most outstanding institution.

### HOSPITALS AND THOSE WHO MAKE THEM.

We have more than once called attention to the interesting portraits of old house surgeons which hang in the board-room of the Royal Infirmary, Sheffield. At the Queen's Hospital, Birmingham, some attempt has been made, we believe, to collect photographs of members of the staff who have done notable work for that institution and for hospitals generally. After the war many hospitals will have added much to their reputation and authority. Every voluntary hospital should have its own historian and a volume recording its history and work. It is a good custom to make the walls of hospital board-rooms gradually to become portrait galleries of worthies. Every such gallery would not fail ultimately to become a centre of interest, local and general. We commend these points as suggestive material for thought and action on their part to the active spirits of every well-administered voluntary hospital throughout the country.

### A KING'S FUND FOR PROVINCIAL CENTRES.

In an interesting report on the extent and ways in which Liverpool charities have been affected by the war, the Liverpool Council of Voluntary Aid comes to the conclusion that, in view of the unequal financial strain thus produced, the war "tends to emphasise the growing need for some substantial central fund in Liverpool, somewhat in the nature of the King Edward Hospital Fund in [*sic*] for London." It would be curious if the war produced central funds in the great provincial cities, for hitherto nothing has been able to do it, though the conditions vary, of course, very greatly in London from those which obtain in the industrial towns. The idea is not a new one. Before the war it was more or less secretly discussed in relation to Birmingham and the Midlands, but, surrounded with vague mystery, it never arrived at the stage of public discussion. How far the views of the Liverpool Council of Voluntary Aid are shared by the Liverpool hospitals we do not pretend to know, but the moral that it draws from its report on existing difficulties is a plain one.

### HOSPITAL ZEPPELIN FINANCE.

Without running any risk of penalties under the Defence of the Realm Act, the well-known fact can be stated that the structure of more than one hospital in this country has been injured by incendiary or explosive bombs dropped by German Zeppelin airships. In one case reference was made by a provincial journal to an unwelcome visit of this character with the equivocal comment, "fortunately the bomb fell on the administrative block," but although the secretary was thus personally attacked, he has not been allowed by a relentless Censor to make capital for his institution out of an undoubtedly pathetic situation. Even months after the misfortune, when the annual report of the executive is being prepared, reference to an incident, probably as well known in Germany as here, was in at least one case peremptorily forbidden. But an accountancy difficulty now presents itself in the case of a hospital receiving compensation through insurance, and expending money on repairs. How to mislead the Teutonic spy searching through the annual reports of the British hospitals to trace accurately the course steered by a particular airship when on its mission of destruction of "docks, Government buildings, and military works," causes deep thought to a conscientious secretary when it comes to having to make clear where part of the institutional income came from and on what it was expended. In one case we notice the difficulty is very neatly met; certain income is shown as "insurance receipts to cover cost of repairs as per contra," and on the other side "costs of effecting repairs covered by insurance as per contra." However, another instance of war-time finance can hardly be described as secretive; here we find as income an amount "received from Lloyd's on account of aircraft insurance," and as expenditure a disbursement described as "partial repairs after incendiary bomb fire." Let us hope that when the next account of this hospital is published, and the full bill for the damage has been met, Censors and Zeppelins will have become of abstract interest.

### NAVAL AMBULANCE TRAIN ON VIEW.

A naval ambulance train, the first to be on exhibition in London, and the latest of its type, will be on view by the public, at a charge of 1s. a head, on Whit Sunday and Whit Monday, in

Addison Road Railway Station (close to Olympia). The train affords an interesting insight, that should not be missed, into the life of the sailors of the Fleet, not only of the Royal Navy, but of the equally heroic Merchant Service of the Empire. The patient is transferred in his cot from ship to hospital, the cot swinging from the roof of the ambulance train during its rail-borne journey. The train contains an operating-theatre, dispensaries, and kitchens, as well as the coaches. The *Dreadnought* Hospital for Seamen, Greenwich, is to receive the proceeds of the exhibition. They could not be given to a more deserving object, for the *Dreadnought*, which never closes its doors to the sick or wounded seaman, is doing a national work in restoring to health the brave men on whose endurance and vigilance in the last resort "the safety," as the well-known phrase puts it, "of these islands does chiefly depend."

### YORKSHIRE AND THE ARC-EN-BARROIS HOSPITAL.

The county of broad acres is taking an especial interest in the English hospital, authorised by the Anglo-French Committee and accepted by the French Government and General Joffre as a military hospital, at Arc-en-Barrois, Haute Marne. The hospital is one of our gifts to France and is staffed by English doctors and nurses. It provides beds for 180 French soldiers, and as it is the nearest hospital to Verdun the beds are never unoccupied. Through the efforts of Miss Eva Moynihan, of Leeds, a Yorkshire bed has been endowed in the hospital; and the town of Keighley has made itself responsible for the maintenance of another bed. Other Yorkshire towns are expected to follow the example thus set.

### THE STAR AND GARTER HOME.

Mr. Arthur Stanley has written to Miss May Whitty, chairman of the British Women's Hospital, asking her committee to raise £100,000 to complete the building and equipment of the Star and Garter Home. In the course of his letter Mr. Stanley acknowledges "the very generous manner in which the British Women's Hospital have met the increased demands which we have made upon them." He adds that the sum of £100,000 will provide "a splendid building, perfectly equipped," and will prove "a magnificent memorial of the devotion of the British women to the men who fought for them in the great war."

### THE PROPOSED RADIUM INSTITUTE FOR BIRMINGHAM.

The Birmingham and Midland Skin Hospital rightly continues to place before the public the needs and ambitions of its radium fund. The former remain constant; the latter have been interrupted, but not deflected, by the war. The proposal still is for the growing electrical and bacteriological departments of the hospital to combine together as an independent unit, which would then be known as the Birmingham and Midland Radium Institute. It is a fact that these departments are out-growing their present accommodation. It is argued that they would prove much more useful than they are at present if they did not confine their observations and work to the consideration of skin diseases only. This idea was hatched in July 1914. The war postponed its realisation; but the fact that it is still exercising the minds of the hospital authorities argues a keen staff, and an administration alert to the advantages to be conferred and derived from the establishment of a special unit of wide scope associated with it. If there is the need for a Radium Institute for the Midlands, as is definitely implied by the supporters of this plan, the idea is comprehensive and ambitious enough to attract the support of the rich and enterprising citizens who have fostered the growth of Birmingham ever since the late Mr. Chamberlain gave them the lead in local affairs.

### DEATH OF THE FOUNDER OF THE AMERICAN HOSPITAL IN FRANCE.

A sympathetic notice in the *Spectator* draws attention to the career and life-work of a very notable American surgeon, Dr. J. W. White, of Philadelphia, who died on April 24. In his native city Dr. White was widely known and respected for his high character and great gifts; but since the war his name has become better known on this side of the Atlantic, and especially in France. Firmly convinced of the justice of the Allies' cause, Dr. White threw himself into American controversies with great energy, combating the German-American propaganda with heart and soul. More than that, he collected funds and gathered together a volunteer staff for the great American Ambulance Hospital which he set up in France. Whenever he was complimented upon the splendid work and equipment of his hospital, Dr. White was wont to reply with emphasis, "It is the *least* we can do for France." In September 1915 he returned to America in the early stages of a spinal complaint which led to his death at the age of sixty-five.

### A LACK OF PATRIOTISM AT THE BELGRAVE HOSPITAL.

The management committee of the Belgrave Hospital for Children, Clapham Road, have applied to the Lambeth Tribunal for the exemption from military service of Mr. T. Chapman, the secretary. It was maintained that special experience and training were required to conduct the hospital, and that men adapted to the work were very scarce. There were no ladies capable of doing the work, and, with all the responsible people away for war purposes, it was essential that the secretary should remain in authority at the hospital. The Tribunal thought that this was work that a lady could well undertake, and they allowed a month's extension to permit the committee to make arrangements so that at the end of that time the secretary could be released. We must say we agree with the Tribunal, and we think the authorities of this hospital ought to be thoroughly ashamed of themselves for attempting to detain a man of military age in the way they did; even if no woman can be found for the post it cannot be impossible to get a man of over military age for it.

### HOSPITAL STAFFS BEFORE THE TRIBUNALS.

On behalf of the General Hospital, application was lately made in the Second Court of the Birmingham tribunal for exemption from military service of various members of the institution's medical, administrative, and engineering staffs. The pleas put forward by Mr. E. P. Beale in support of these applications were based upon the fact that all the beds were generally occupied, and that on the surgical side at least the work was heavier than usual. It was also pointed out that in place of the fourteen qualified medical men on the hospital's staff who figured there in normal times, there were now eight qualified and six unqualified men; out-patients had also to be dealt with. In the event exemption was granted so long as the men continued to hold their present positions, except in the case of two clerks, who received temporary exemption only.

### THE TAXPAYERS VERSUS THE DISCHARGED SOLDIER.

Our leading article in The Hospital of May 27, entitled "Shall we Sweat the Crippled Soldier?" which warned the public not to permit the pensions of the permanently disabled to be reduced by mulcting them of anything they might be able to earn from freak activities, happily coincided with a stirring speech made by Sir Frederick Milner on another aspect of the same subject. At the annual meeting of the Incorporated Soldiers' and Sailors' Help Society on May 31 Sir Frederick Milner said that the scandals which occurred after the South African war must not be repeated. He urged the establishment of a new national pension committee in place of the "sort of stop-gap" set up by the Government, with wide powers and no red tape. The disgraceful fact that, at the end of twelve months of warfare, 15,500 men had been discharged from the Army without a penny of pension had, he said, been since largely remedied. The present Pensions Committee, the Chelsea Board, was too old-fashioned and too much choked with red-tape. In the case of a man named Wells, discharged from the Navy after contracting double pneumonia, 6d. a day for one month was given, and on the day on which the Admiralty increased this sum to 17s. a week the man died—at the employment to which necessity had sent him. "I told Mr. Balfour," Sir Frederick remarked, "that the death of this man lay at the door of the Admiralty."

### HOSPITAL WORK AT THE CAPE.

Of the fact that the Dominions across the seas as well as the Motherland have special war troubles in respect of hospital work we are reminded by the annual report of the Cape Hospital Board. It was to be expected that the proposed programme in continuation of the unification scheme detailed in last year's report could hardly proceed exactly as in the piping times of peace. The institutions now controlled by the Board comprise five hospitals, the largest being the Cape Town Somerset Hospital, with 225 beds, one dispensary, two convalescent homes, and a district nursing organisation. The total cost of the work of these institutions in 1915 was £47,995, only about 11 per cent. of which sum came in the form of voluntary contributions, and just over half was provided by Government grants. A system of graded payments by patients is, we note with pleasure, scientifically and successfully carried out, bringing no less a sum than £13,203 into the hospital treasury. One of the chief effects of the war has been the suspension of many items of the building programme. The contracts for annual supplies and services to the group of institutions have been centralised, but under the extraordinary condition of markets due to the war, opinion is withheld as to whether the new system is more advantageous than the previous arrangement under which each institution contracted separately.

### CALCUTTA HOSPITAL FOR TROPICAL DISEASES.

It is usually the case that the foundation of a new school of medicine is in connection with an established hospital; but in Calcutta, where, in 1914, a School of Tropical Medicine was inaugurated, the order has been reversed, and the building of a Hospital for Tropical Diseases has followed the foundation of the school, an adjoining site having been provided by Government. On February 24 the foundation-stone of the new institution was laid by Lord Carmichael, the Governor of Bengal, and on another page will be found the plans of the new building, a three-storeyed structure which will cost three and a-half lakhs of rupees (about £23,000) to build and equip. The new hospital starts under the best auspices, for no one can challenge the wisdom and desirability of collecting the different cases of tropical diseases under one roof and in proximity to the appropriate teaching centre. The funds of the new hospital have been chiefly raised by Sir Leonard Rogers and Sir K. C. Bose, whose names are also largely associated with the foundation of the school. In the list of contributions the name of H.H. the Maharaja of Nepal is noticed as having given 10,000 rupees, and at the ceremony it was announced that the Rockefeller International Health Commission of New York had offered an annual grant in order that the subjects of protozoology and entomology may be divided between two professors instead of being in the hands of one, as at present arranged. A special feature is the provision of fourteen small separate rooms for private patients, who will contribute to the upkeep of the hospital. This is the first important hospital to be built entirely by public subscriptions in India with educational and research functions.

### INFANTILE MORTALITY.

The wisdom of the London County Council in assuming control of lying-in homes, in spite of the demand from the Borough Councils for local control, is proved by a report of the London County Council Public Health Committee regarding infantile mortality. The Committee was asked to consider and report whether it were possible and desirable for the Council to take any steps to ensure that adequate measures shall now be taken by the local health authorities more effectively to diminish infantile mortality and suffering, as recently suggested by the Local Government Board. The Committee now reports that the only powers exercisable by the Council which relate to the welfare of mothers and infants are those contained in the Children Act 1908, the Midwives Act 1902, and the new powers regarding lying-in homes. The suggestions of the Local Government Board relate to the provision of schools for mothers, children's welfare centres, and the appointment of health visitors, powers with respect to which are exercisable by the Borough Councils. The London County Council, therefore, has no power to deal with such matters. The Committee, nevertheless, is of opinion that, in view of the need for preserving infant life, particularly having regard to the mortality arising from the war, it is essential that the powers of the Borough Councils should be utilised to their fullest extent. Such powers are not so utilised at the present time, and hence the Committee deems it desirable that the attention of the Local Government Board should be drawn to the matter, with a view to a comprehensive scheme for maternity and child welfare being established throughout London.

### LUPUS IN SCHOOL CHILDREN.

The value of the medical inspection of school children is shown by the numbers of early cases of lupus which are now sent to hospitals for treatment by the Finsen light, while their cure is still a comparatively easy matter. That lupus is being recognised and treated in practically every case in children of school age before disfigurement has become permanent is a proof of the valuable service that is being done by school doctors and nurses. There is no doubt that the careful sifting of cases of lupus and other conditions likely to disfigure or interfere with the development of the school child and their immediate treatment cannot fail to have far-reaching effects for good not only on the physique but upon the happiness of many men and women of the working classes in the years that are to come. Facts and experience gained in this field of nursing will be welcome if sent to the Editor by workers in the school clinics and light departments throughout the country.

### VICARIOUS ECONOMY.

By substituting margarine for butter and reducing the officers' weekly allowances of meat from six pounds to four and of milk from seven pints to six, the Pontefract Board of Guardians hopes to economise to the extent of £300 a year. We have nothing to urge against the substitution of margarine, and we cannot say whether the reduction in the meat and milk is justifiable, because we have not the whole of the dietary scale before us. It may be pointed out, however, that the officers receive rations as part payment for their services. The dietary was either wastefully extravagant in the past or the officers are now being required to give up part of the remuneration due to them. If the former was the case, the Guardians stand condemned. If the latter view is correct, and the staff is voluntarily giving up part of its remuneration for patriotic reasons, it is entitled to full credit for the sacrifice made; but the Guardians have no right to require this sacrifice without the consent of the officers concerned.

### AN OPPORTUNITY FOR DONCASTER.

Miss Beckett Denison, of Doncaster, whose death occurred on May 24, was a very generous supporter of the Doncaster Infirmary. Owing to the opening out of collieries in all directions round Doncaster, the present infirmary has become inadequate, but the committee was unable to make any move on account of the expense. It is now announced that Miss Denison has left her large residence, with its extensive grounds, as a site for a new infirmary. This is an important fact, and we hope the hospital authorities will take care to secure the services of a really competent adviser, so that the scheme of construction of the new hospital may be up to date and full of interest.

### TUBERCULOSIS ACTIVITIES IN THE MIDLANDS.

Notwithstanding some interruption of its activities owing to the war considerable progress has recently been made by the Staffordshire, Wolverhampton, and Dudley Joint Tuberculosis Committee. Temporary dispensaries have been opened at Biddulph, Cheadle, and Leek, and a site obtained for a joint hospital and dispensary at Newcastle-under-Lyme. At Stafford a permanent dispensary has been erected, and premises acquired at Uttoxeter for a sub-dispensary. Plans for dispensaries which are to be provided at Wednesbury and Dudley have been approved, but actual building operations are deferred until after the war and temporary premises now used. The number of persons treated at the several dispensaries varies between 500 and 800 a month. No steps have been taken, owing to the war, with regard to the erection of a sanatorium on the site acquired at Lower Penn, but Groundshow House, Tittensor, has been opened as a sanatorium for twenty patients.

### THE PREVENTION OF MEASLES.

The greatly increased attention that is being paid to measles as a source of child-mortality and of national disablement is reflected by the allocation of two Chadwick lectures to this topic, delivered at the Royal Society of Medicine by Dr. Joseph Cates, medical officer of health of St. Helens. For the most part the lecturer, quite rightly, travelled over familiar ground in his account of the ravages caused by this disease and in his suggestions for mastering it. We do not know how large an audience the Chadwick lecturers usually address—possibly not very large ones; but the management of the Trust very properly take steps to ensure a good deal of publicity for the lectures themselves, whereby a larger public is reached. Perhaps the feature of Dr. Cates's address most likely to impress a lay audience and to help on reform was his reference to the five or six Government Departments which direct the energies of sanitary authorities, education authorities, boards of guardians, insurance committees, and after-care committees; and to the need for co-ordination between them. In other words, the establishment of a Ministry of Health is clearly indicated as the remedy for the confusion and overlapping which now exist.

### THE INCINERATION OF HOUSEHOLD REFUSE.

A HEALTHY form of compulsion will be applied to householders if the Wallasey local authority has its way. Repeated appeals have been made to the Wallasey public, by means of speeches in the council chamber and by pamphlets circulated broadcast, to burn their household rubbish instead of depositing it in the dustbin or elsewhere. The response has not been satisfactory, and the medical officer's warning as to the serious menace to health which the present system entails has passed almost unheeded. The health committee, realising the gravity of the position brought about by the shortage of labour, has resolved upon drastic lines of action, and is asking the Local Government Board to give it power to compel householders to burn their refuse during the war.

### THE FLIGHT OF A PATIENT.

WE are all familiar with escapes from asylums, but we imagined that the public conscience had been sufficiently quickened to prevent an isolation hospital from being regarded as a prison, unless, of course, persecution or ill-treatment were alleged. It appears, however, that this view is too sanguine, since a patient, a young man, accommodated in the scarlet fever block of the Sedgefield Isolation Hospital, lately took advantage of his convalescence to escape. The committee's report records that on the evening in question the night nurse was called away to an urgent case in another part of the block, whereupon the young man disappeared. The grounds were searched in vain, but he was eventually tracked to his home, brought back, and reprimanded for his conduct by the deputy medical officer, Dr. Bazan. The parents were asked to refund the cost of the cab. It is a pity that the committee's report on the affair, as published, contained no reference to the young man's motive. Presumably he thought that he was being detained too long. But it would be instructive to learn the real motive which prompted his escapade. We should also like to know what steps were taken to disinfect the cab.

### THE "SOUTHERN CROSS" IN JUNE.

THE current issue of the journal of the 1st Southern General Hospital, Birmingham, again has some pleasant line illustrations, but its most curious feature is the following paragraph, contributed by "C. L.," which we transcribe in full as a curiosity among epitaphs: "There exists in the graveyard of St. Mary's Church, Stafford, a curious inscription recording a surgical operation, as follows: 'Sacred to the memory of William Hanbury, in the county of Stafford. He died in the General Infirmary the 6th day of March (18)12 Aged 63 after an operation by which a stone was extracted from the Bladder of the size of under.' Then follows a diagram of Mr. Hanbury's stone, which local wits have since enlarged until it measures 7 in. by 3½ in., and forms a very respectable-sized puddle in wet weather." Above this paragraph is an ambitious and interesting cartoon, all the better for not being farcical, showing "John Bull asleep in the garden of tradition." The articles "Torpedoed" and a "Battle in the Air" give apparently first-hand accounts of two exciting experiences. The "Who's Zoo" cartoon is again both decorative and ingenious.

### AN EPSOM HOSTEL.

THE Rev. T. Gage Gardiner, Rector of Lambeth, is appealing for funds for a Mothers' Hostel started six months ago at Epsom for young unmarried mothers and their babies. A house in three acres of ground together with a well-stocked orchard has been taken at Epsom, and will accommodate twenty mothers with their babies. Varied methods of training and employment are provided; market gardening, poultry-keeping, and upholstery work, besides ordinary domestic service, are among the subjects taught. During the period of training the cost of maintenance of mother and child is estimated at £15 a year; presumably this means that the hostel is partly self-supporting, and that the deficit on its working amounts to that sum per patient. Guarantors, of whom Mrs. Randall-Davidson is one, have undertaken to defray half the cost of maintenance for three years. It will be interesting to watch the development of this well-conceived charity, which, properly managed, should have a great future before it. The honorary secretary of the Hostel is Mrs. de Wesselow, Ebbisham House, Epsom.

### THIS WEEK'S DRUG MARKET.

ON the whole business has been quieter during the week. Many of the price changes have been in a downward direction, among the drugs which share this tendency being cocaine, acetanilide, antipyrine, amidopyrine, cod-liver oil, salicylic acid, sodium salicylate, citric acid, tartaric acid, salol, and sulphonal. It seems quite probable that the downward movement in the price of cocaine has not reached its limit, and buyers would perhaps be well advised to restrict their purchases to their immediate requirements. There are rumours of an impending boom in quinine, but at present there is apparently nothing to support this rumour. A good business has been done in formaldehyde, and as supplies are scarce prices tend upwards. Bromides continue very quiet, but prices are unchanged. Phenacetin and barbitone are both appreciably dearer, and trional, owing to scarcity, tends upwards in price. After a fair business in potassium permanganate at lower rates prices are higher again. At the recent public sale of drugs in Mincing Lane substantially higher prices were paid for Jamaica honey; very high rates were paid for senna leaves; Cape aloes, ipecacuanha, Japanese peppermint oil, and rhubarb had a lower price tendency.

